# Positioning Information Privacy in Intelligent Transportation Systems: An Overview and Future Perspective †

**DOI:** 10.3390/s19071603

**Published:** 2019-04-03

**Authors:** Aleksandr Ometov, Sergey Bezzateev, Vadim Davydov, Anna Shchesniak, Pavel Masek, Elena Simona Lohan, Yevgeni Koucheryavy

**Affiliations:** 1 Tampere University, 33720 Tampere, Finland; elena-simona.lohan@tuni.fi (E.S.L.); yk@cs.tut.fi (Y.K.); 2 Saint-Petersburg State University of Aerospace Instrumentation (SUAI), St. Petersburg 190000, Russia; bsv@aanet.ru; 3 ITMO University, St. Petersburg 191002, Russia; vadim.davydov@niuitmo.ru (V.D.); anna.schesnyak@scaegroup.com (A.S.); 4 Brno University of Technology, 60190 Brno, Czech Republic; masekpavel@vutbr.cz

**Keywords:** Intelligent Transportation Systems, positioning, data privacy, authentication, GDPR

## Abstract

Today, the Intelligent Transportation Systems (ITS) are already in deep integration phase all over the world. One of the most significant enablers for ITS are vehicle positioning and tracking techniques. Worldwide integration of ITS employing Dedicated Short Range Communications (DSRC) and European standard for vehicular communication, known as ETSI ITS-G5, brings a variety of options to improve the positioning in areas where GPS connectivity is lacking precision. Utilization of the ready infrastructure, next-generation cellular 5G networks, and surrounding electronic devices together with conventional positioning techniques could become the solution to improve the overall ITS operation in vehicle-to-everything (V2X) communication scenario. Nonetheless, effective and secure communication protocols between the vehicle and roadside units should be both analyzed and improved in terms of potential attacks on the transmitted positioning-related data. In particular, said information might be misused or stolen at the infrastructure side conventionally assumed to be trusted. In this paper, we first survey different methods of vehicle positioning, which is followed by an overview of potential attacks on ITS systems. Next, we propose potential improvements allowing mutual authentication between the vehicle and infrastructure aiming at improving positioning data privacy. Finally, we propose a vision on the development and standardization aspects of such systems.

## 1. Introduction

Today, the technology is penetrating most of the modern digital systems [1]. Billions of interconnected devices are already deployed, and many would join them soon in the race towards smart interconnected world [2]. One of the promising paradigms is the utilization of Intelligent Transportation Systems (ITS), which is driven by one of the biggest markets being automotive [3]. The technologies covered by ITS are usually split into two major groups: vehicle-to-vehicle (V2V) [4,5] and vehicle-to-infrastructure (V2I) [6,7]. In an attempt to cover the challenges from both groups, a new trend called vehicle-to-everything (V2X) [8,9] has recently emerged, focusing on interconnecting cars with other surrounding objects.

Different standardization communities are already working hard to standardize the technological requirements, thus aiming for the same goal: to develop a unified ecosystem that would allow reliable, fast and secure communications between vehicles and roadside infrastructure. Such technologies as Dedicated Short Range Communications (DSRC) in the USA [10,11] and ETSI ITS-G5 in Europe [12,13] are actively developing aiming at being deployed in the oncoming decade. Moreover, DSRC has recently been selected as a V2X communications technology in the USA which means that all the newly produced cars would be equipped with a wireless IEEE 802.11p module [14] that could also be used for positioning tasks [15]. Japan is also actively involved in equipping the vehicles with DSRC aiming to have more than 100,000 operational by 2025 [16].

Previously, V2X development was not so widespread as smart cars were limited in numbers, highly priced and thus inaccessible to the majority of the world’s population. In this paper, we will focus only on the vehicular positioning privacy in an urban scenario where a conventional Global Navigation Satellite System (GNSS) can be affected negatively by propagation in complex environments issues [17]. Nowadays, this problem becomes exceptionally significant due to the fast development of vehicle use delegation [18] in the car-sharing business [19,20].

Indeed, GNSSs proved themselves to be inefficient in urban-canyon operation scenario [21] also known as multipath interference phenomenon, caused by tall buildings blocking lines of sight (LOS) from the receiver to the navigation satellites. Some works have proven that no-line-of-sight (NLOS) signals could still be used for positioning if longer integration times and data wipe-off are present [22,23].

Many solutions were proposed to mitigate the challenges of efficient vehicle location determination. Simultaneously, operation in the city allows broad communication possibilities due to high coverage of various wireless technologies including conventional cellular and other heterogeneous techniques [24,25].

Overall, several radio-ranging-based cooperative positioning (CP) techniques were already proposed to enable vehicular localization in urban environment [26]. Nonetheless, the localization problem in conventional Mobile ad hoc networks (MANETs) with range measurements is often tackled by trilateration and multilateration to some fixed or mobile anchor nodes [27].

The internode distance is commonly measured using radio-ranging or range-rating techniques such as the time of arrival (TOA), time difference of arrival (TDOA), received signal strength (RSS), and other techniques [28]. The infrastructure in ITS provides the nodes not only with precise positions on the trusted infrastructure units but could also deliver regularly updated maps of the RSS based on the devices involved. Despite common V2I infrastructure nodes, the development of cellular networks beyond 5G would allow more precise positioning by utilizing specific synchronization signals with 5G New Radio (5G NR) even under high mobility constrains [29].

Since vehicular positioning mostly relies on the data provided by the Location Solution Provider (LSP), many concerns arise in regard to controlling personal information of the user [30]. Potential misuse of such sensitive data by LSP while user blindly agrees to the terms of use or even unauthorized tracking of the vehicle may occur when relying on the ITS positioning techniques. Note, that such situations appear even today and many security-related questions of said networks are still open [31]. For example, U.S.-based LocationSmart company was leaking the sensitive information about the positions of the smartphones operating under various cellular operators [32]. A similar case was reported earlier the same month by The New York Times, the service initially used to monitor calls from inmates and allowing to find almost any cell phone in the U.S. in seconds was misused [33]. Note, it is possible to uniquely identify the node based on just four hours of the monitoring while having access to the position-related data provider in 95% cases [34] based on said digital activity ‘footprints’ [35].

From the V2X positioning perspective, researchers have proposed a considerable number of privacy-preserving protocols for data exchange between the vehicle and the surrounding nodes [36]. Most of these protocols imply authentication of the vehicle to the trusted anchor nodes [37]. However, protocols without mutual authentication may be vulnerable to a wide range of attacks that should be carefully taken into consideration during the system development phase. The main contributions of this paper can be listed as:

1. an overview of existing privacy-related V2X solutions for infrastructure-based ITS systems;
2. a modified solution for data privacy enhancement based on well-known protocol;
3. a discussion of possible cybersecurity attacks on mentioned systems;
4. an overview of present standardization and General Data Protection Regulation (GDPR) related activities.

The rest of the paper is organized as follows. First, we survey the existing solutions for vehicular localization in Section 2. Next, we provide an overview of the protocols that utilize additional data from the environment/user to assist in locating the vehicle in Section 3. Further, we propose a simple extension to said protocols in Section 3 and Section 4. Next, we elaborate on potential attacks on proposed architecture in Section 5. The last section provides future perspectives concerning standardization aspects and concludes the paper.

## 2. Solutions for Spotting of Vehicles on the Road

**Figure 1:**
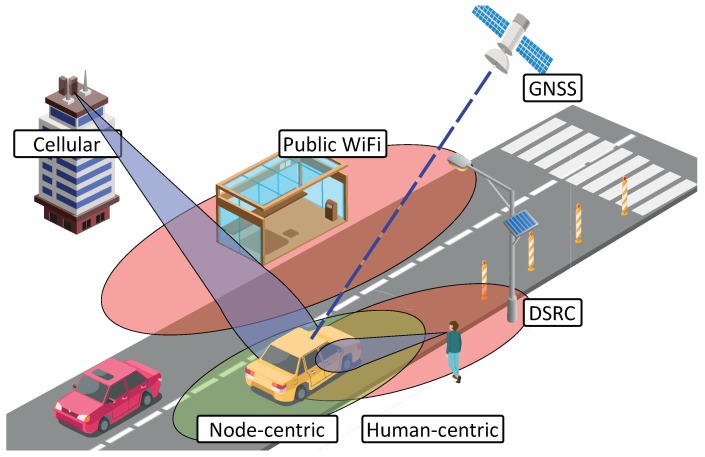
General example of vehicular positioning techniques.

In this section, we consider several approaches for the localization techniques and the corresponding benefits and drawbacks. First, we list various potential ways to determine vehicle location with a simplified representation given in Figure 1.

### 2.1. Global Navigation Satellite Systems

One of the GNSSs is the Global Positioning System (GPS) which is a technology, network, and service owned and maintained by the USA [38]. The GPS service provides end-users with an opportunity to estimate their position and to retrieve globally synchronized time. Entire GPS architecture consists of space, control, and user segments [39]. The space segment is comprised of a constellation of 31 satellites (as of March, 2019), not including the decommissioned, on-orbit spares. GPS satellites operate in medium Earth orbit (MEO) at an altitude of approximately 20,200 km (11,550 miles) [40]. The GPS control segment is comprised of a global network of ground stations designed for tracking the constellation of satellites in MEO and also monitoring their transmissions, communicating and analyzing the data. The user segment consists of end users (civilian and military).

The second GNSS giant is the Russian Global Navigation Satellite System (GLONASS) which serves the same role despite some minor differences [41]. GLONASS satellite orbits are arrayed in three planes, separated from one another in right ascension of ascending node by 120 degrees, with eight satellites in each plane. The number of spacecraft is 26 including 24 actively operational ones (as of March, 2019), and the altitude is 19,100 km (11,868 miles) [42].

Finally, GNSS positioning technology widely used in the European Union is GALILEO starting 2005 [43]. GALILEO system currently has 26 operational satellites on the orbit placed in 3 orbital planes, at 29,600 km (18,400 miles) altitude [44]. Worth to note, the GNSS system specifically utilized in China–BeiDou Navigation Satellite System (BDS). BDS constellation currently has 17 operational satellites, and the number is expected to reach 35 by 2020 [45]. Nonetheless, most of the mass-produced vehicles are already equipped with both GPS and GLONASS systems [46].

The fundamental principle of using the GNSS system is to determine the location by measuring the timing of the reception of a synchronized signal from the navigation satellites [47,48]. Despite almost-the-entire-globe coverage, GNSS has a number of challenges that could not be neglected.

To start with, the material penetration characteristics limit the use of GNSS in hard-reach places such as underground parking, tunnels, etc. Next, because of the near-spherical shape of the Earth calculating an accurate distance between two points requires the use of spherical geometry and trigonometric math functions. However, many applications calculate an approximate distance using simplified ones. Thus, the GNSS-based calculation of the distance between two objects can lead to errors of 10 percent or more [49]. The most significant issue of the GNSS is its propagation in urban environments where multipath fading has a tremendously adverse effect on the position estimation.

### 2.2. Infrastructure-Based Methods

Some vehicles are already equipped with conventional IEEE 802.11 wireless modules commonly referred to as Wi-Fi. By using previously estimated maps together with sensed Received Signal Strength Indicator (RSSI) along with the Base Station (or access point) Identificator (BSID), the vehicle can approximate its location in the complex metropolitan environment, which is generally a challenging task for GNSS [50]. This methodology would be widely used in DSRC since all of the infrastructure nodes positions would be known forming a full map of BSIDs [51].

The main drawback of the technique is the need for continually updating the RSSI maps since the changes in the environment (such as new roads, new trees, new buildings, etc.) affect the RSSI, and Wi-Fi is operating in unlicensed spectrum affected by many interference sources. Therefore, the Wi-Fi network faces changes over time, and these changes need to be monitored continuously in order to rely on Wi-Fi positioning. Nonetheless, another drawback of using Wi-Fi for vehicular positioning is the lack of centralized infrastructures and the difficulty to achieve a high quality of positioning system, due to significant fluctuations in RSS of Wi-Fi APs. The advantage of using a Wi-Fi network is the possibility to serve in the metropolitan scenario with complex GNSS propagation characteristics, where GNSS is likely to fail. Similar issues could be encountered while utilizing any short-range wireless technology, such as Bluetooth Low Energy (BLE) [52] or Zigbee [53].

The other technique that could be utilized by smart cars is so-called cellular-positioning that has been used for localization of phones over the decades. Already in 2G, the Cell-ID-based positioning with accuracies on the order of a few hundred meters was feasible [54]. The efficiency was improved to tens of meters in 3G using TDOA measurements. Current cellular based-solutions also make use of Angle of Arrival (AOA) or combined AOA-TOA techniques [55].

### 2.3. 5G Communications as an Improvement for Positioning

The evolution of wireless communications brought us to the next frontier of positioning as well [30]. Moving towards higher millimeter wave (mmWave) frequencies (>6 GHz) as part of 5G NR systems networks pushes towards the utilization of highly directional antennas on both transmitter and receiver sides [56]. In mmWave frequencies higher than 30 GHz, the lower signal wavelength would allow for packing hundreds of antenna elements in a small area, enabling the implementation of highly directional beamforming capabilities, thus providing better spatial reuse and, consequently, better positioning [57]. In particular, it is achieved by utilizing massive Multiple Input Multiple Output (MIMO) technique [58]. 5G positioning for vehicular networks represents its separate niche or interest [59]. 5G NR is generally expected to provide an accurate positioning from a single reference base station (BS) also form multipath components of up to 0.5 m [60], which is especially beneficial for V2I scenarios.

### 2.4. Node-Centric Localization

This method enables non-instrumented vehicles to determine their locations by collecting data from neighboring vehicles through V2V direct communications, Light Detection and Ranging (LIDAR) techniques, radars, etc. The node-centric approach specifies the routing path as a sequence of connected nodes. Every vehicle has an opportunity to communicate and sense some or all of its neighbors as depicted in a Fog Internet of Vehicles paradigm [61].

As an example, to localize a vehicle among its neighbors, the authors in [62] propose a distributed algorithm that uses inter-vehicle distance estimates obtained via a radio-based ranging technology. There are two kinds of nodes involved, namely common nodes and beacon nodes; beacon nodes can determine their location, whereas common nodes are not location-aware. The localization process is based on the estimation of common nodes locations. This method is beneficial for hard-to-reach places, e.g., tunnels, underground parking. In [63,64], and in [65] the technologies of cooperative driving with automated vehicles and intervehicle communications are shown along with the corresponding benefits.

Despite actual communication devices utilizing mmWave band, radars open another prospect for vehicle localization, especially significant for autonomous driving since most of the vehicles of this type are already equipped with some LIDARs and radars. Trailblazing work [66] shows the benefits of utilizing a combination of mmWave radar together with static beacons as an indistinguishable replacement of the GNSS system. Authors in [67] proposed and proved that utilizing omnidirectional mmWave radar allows to reach 25 cm-level accuracy even under the effects of snowfall.

### 2.5. Human-Centric Localization

Human-centric localization is targeted at people carrying gadgets [68,69]. As soon as a person starts to approach the vehicle, a gadget (smartphone, smartwatch, or augmented reality glasses) is changing the state to pre-authentication phase [70]. From telecommunications perspective, the enablers for such authentication are already present in current 3GPP LTE deployments [71] and ProSe service responsible for handling those as part of Device-to-Device (D2D) communications [72]. Therefore, the preliminary connection establishment could be automatically detected by the vehicle, and the last known location of the hand-held device could be utilized to improve the positioning of the vehicle.

There have been numerous studies focusing on human-vehicle interaction ways of driver identification using sitting postures were investigated in [73], the interaction between a human and a vehicle is described in [74], and authors provide an overview of the importance of human-vehicle interaction in autonomous vehicles in [75].

As positive aspects, we can distinguish the high accuracy in location determination owing to the availability of base station cell IDs and, consequently, the availability of data for calculation of the approximate vehicle location [76]. On the other hand, this method is highly dependent on the actual availability of devices, e.g., device utilization might become impossible due to insufficient battery power.

### 2.6. Verifiable Multilateration

Generally, verifiable multilateration is a technique based on the measurement of the difference in distance between two or more stations at known locations by broadcast signals at known times. Recently, researchers have proposed a number of positioning and distance estimation techniques in [63,77]. This method requires to install a set of base stations controlled by a central authority, for example, a cellular or Low-Power Wide-Area Wireless Technology (LPWA) operator. If the base stations can uniquely compute the vehicle location using these distance bounds, and if this location falls into the triangular pyramid formed between the verifiers, then they conclude that the vehicle location is correct. Equivalently, only three verifiers are needed to define the vehicle location in two dimensions; the verifiers still consider the location correct if it can be uniquely computed and if it falls in the triangle formed between them [63].

This approach poses a number of problems, namely:

- Simultaneous reception: In this method, the vehicle communicates with at least three base stations in order to obtain its current coordinates. Certainly, in the real world, it is impossible to get three signals perfectly synchronized.
- Security: In the communication process, it is vital not to allow the attacker to receive the transmitted data. In [78,79] the potential cyber-attacks specific to automated vehicles are investigated. Therefore, it is necessary to provide a secure transfer of information between the station and the vehicle.
- Confidence or trustability of the access nodes: Before transmitting the information, it is required to ensure that the base station is trustworthy valid by employing mutual authentication. Some of the approaches to mutual authentication were described in [80,81]. This problem could also be addressed with conventional role-based models [82].
- Anonymity: In some situations, the base station is not supposed to obtain any information about the vehicle, neither the identification nor the location. In this paper, we show several protocols fulfilling mobile node anonymity requirement.

## 3. Vehicle Location Protocols Using Additional Information

Previously listed methods allow for the design of an effective hybrid system meeting the necessary security requirements and being both accurate and fast in determining the location [83]. A number of experiments have already been carried out, proving the benefits of such utilization [84,85].

**Figure 2:**
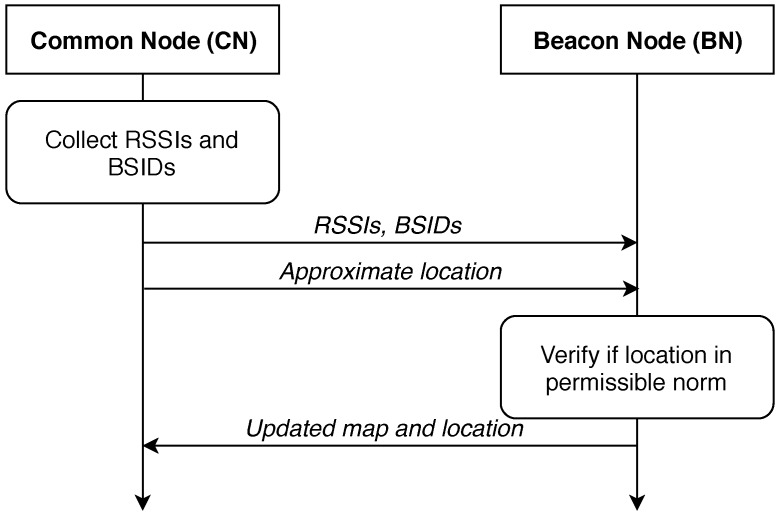
An illustration of communication between common and beacon nodes.

The grouping could be done as following. As for the first strategy, we assume having ‘beacon’ and ‘common’ nodes in the system. The modification is such that the beacon node is a node where a passenger with a smartphone is present. Furthermore, the smartphone can identify the location more accurately due to cellular signal presence. Common nodes are the nodes where the smartphone does not transmit any information about the location. In this case, the beacon nodes are polled to receive it. At the same time, all vehicles monitor their surroundings for infrastructure units and corresponding RSSI values. The protocol execution example is shown in Figure 2 for a beacon node and a common node.

A beacon vehicle has a GNSS receiver and a list of available infrastructure access points (APs). At this moment, a common node is location-unaware. Hence it requests the beacon node for a list of available infrastructure APs. In the reply, the beacon node also transmits an approximate location in addition to requested data.

Indeed, the positioning techniques as we know them today are relying on their own collected location data. The systems of tomorrow would also rely on neighbors and infrastructure thus moving the information security aspects to an entirely new level.

In this section, we consider possible protocols for data exchange between a vehicle not possessing reliable location information and its surrounding roadside elements and other traffic participants carrying location data. The crucial feature of these protocols is the presence or absence of anonymity of the vehicle requesting location information. The anonymity of the request for location is an essential property of the protocol, and its availability requires significant complication of the protocol. The anonymity in this work is considered as the process of ‘hiding’ the actual vehicle location. Generally, the localization process could be viewed as follows.

- First, the ‘indirect’ distance to the static trusted nodes obtained from the known units (cellular or infrastructure units) is estimated. Mutual authentication also takes place during this phase.
- Next, the distances are utilized to estimate the location of the node through classical geometry by, for example, triangulation.

We further list the potential options for securely executing the first phase.

### Distance Determination without Anonymity

**Figure 3:**
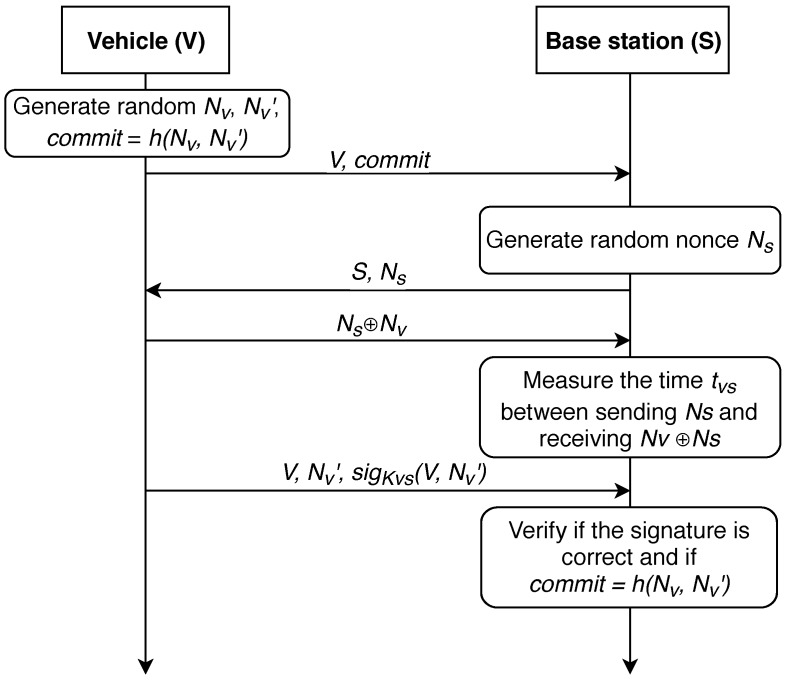
The distance-bounding protocol P-04 [86].

In 2004, authors proposed a distance-bounding protocol in [86], we further refer to it as protocol P-04. The action here is performed between *V*—the vehicle, and *S*—the base station. Key constructs utilized by this work are given in Table 1. The protocol is shown in Figure 3.

First, a shared symmetric key must be generated between the vehicle and the infrastructure node for secure exchange of information. Key generation algorithm depends on the computing power modules installed in the vehicle and base station. Now, it is presumed that such a link has already been established and the data exchange between the station and the vehicle takes place via the secure medium. It is also presumed that a shared symmetric key was delivered after the devices have established a secure link employing, for example, the well-known Diffie-Hellmann protocol. Thus, common pairwise key $K_{VS}$ could be utilized for the message authentication for both vehicle and BS. Basically, we state that correct deciphering of $e=E_{K_{VS}}\left(m\right)$ and obtaining *m* is only feasible for the vehicle and the BS.

The first step of the protocol for determining the distance between the base station and the vehicle is the generation of two random nonces ($N_{V},N_{V}^{\prime }$). Next, it in necessary to calculate their hash function *h* (commit) on the vehicle side and to send the result to the infrastructure. In turn, the BS generates one nonce ($N_{S}$) and sends it to *V*. The vehicle must calculate $N_{S}$⊕$N_{V}$ and return it.

The key point of the protocol is that *S* measures the time between sending $N_{S}$ and receiving $N_{S}$⊕$N_{V}$. Using this time, the infrastructure can estimate the distance $d_{VS}$ to the vehicle. After that, *V* communicates with the base station, sending the identification number id, $N_{V}^{\prime }$ and the signature of id and $N_{V}^{\prime }$, using their pairwise symmetric key $K_{VS}$. Base station *S* verifies if the signature and $commit=h(N_{V},N_{V}^{\prime })$ are correct. If all checks are correct, the base station considers the distance $d_{VS}$ computed at the previous step to be reliable. Further, the received value $d_{VS}$ can either be sent to the vehicle or used on a dedicated server to calculate the vehicle’s location.

**Figure 4:**
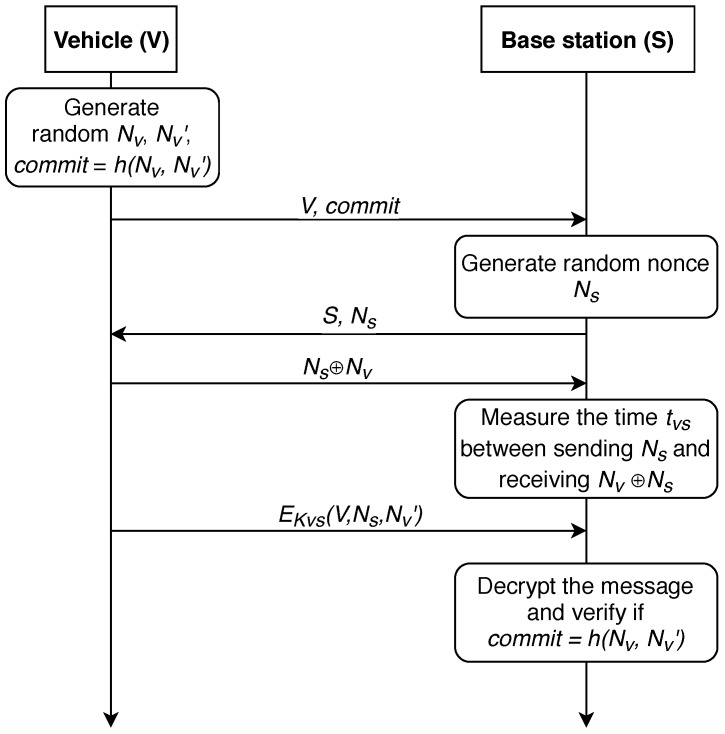
An improved distance-bounding P-06-M protocol.

In 2006, this protocol was improved in [63], further referred as P-06 protocol. The modified strategy is shown in Figure 4. In this improved version of the protocol, only the last two steps were modified. *V* sends encrypted information, its id number, $N_{S}$ and $N_{V}^{\prime }$ with the symmetric key $K_{VS}$. *S* decrypts the message and verifies if $commit=h(N_{V},N_{V}^{\prime })$.

**Figure 5:**
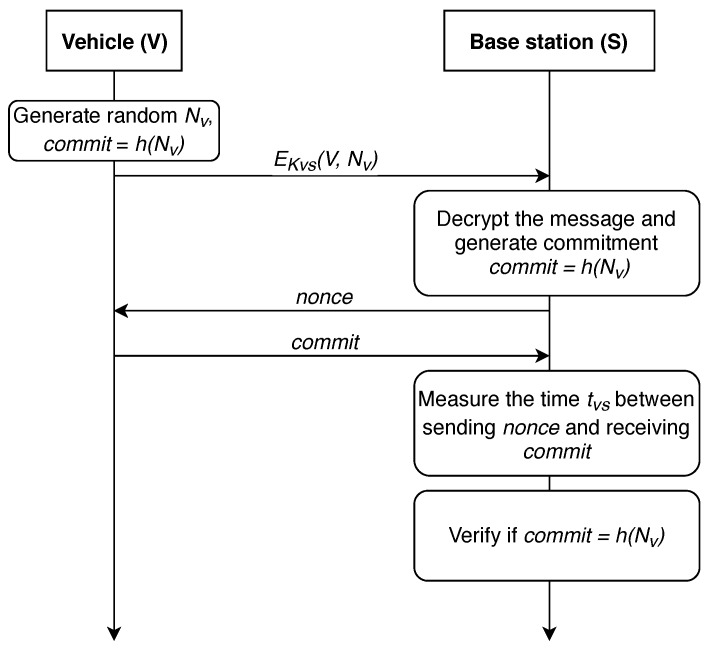
Potential improvement of distance-bounding protocol.

Carrying out a XOR operation imposes extra computational cost; to address this problem, we propose to modify the protocol slightly. Proposed modification is shown in Figure 5 and detailed in [87].

This modification eliminates the necessity for XOR calculation. Should both the vehicle and the infrastructure node require to determine vehicle location, an extra step can be added to the protocol, namely, as $S\to V:E_{K_{VS}}\left(t_{VS},N_{V}\right)$.

The above-listed protocols assume that the vehicle is a location-unaware data transmission initiator. We note that discussed protocols suggest vehicle authentication via Public Key Infrastructure, while mutual authentication is not present. The protocols should be improved to allow mutual authentication since a malicious BS attack could be executed, thus compromising the localization process. We propose our improvement in the next section.

## 4. Location Determination with Mutual Base Station Authentication

**Figure 6:**
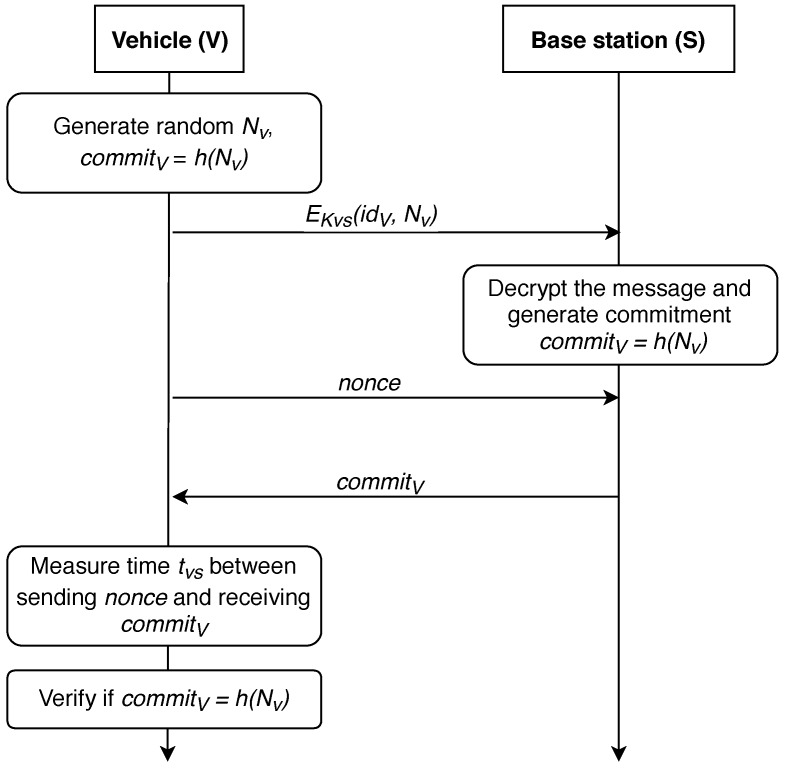
Distance-bounding protocol with base station authentication.

Next, we consider the modification of the described protocol that allows for ensuring the anonymity of the vehicle while determining the distance to the base station, further referred as P-AF-BS protocol and shown in Figure 6.

In the previous section, we discussed the protocols for vehicle-to-base station communication by transferring the corresponding vehicle id and location, yet this approach is vulnerable to a number of security issues. An attacker controlling a malicious BS could gain access to the localization of any vehicle, control its movement, or deceive the vehicle by transmitting the wrong distance, which might lead to unpleasant consequences. Security of such a protocol might be significantly improved by not allowing the base station access to precise vehicle location. There are two possible ways of delivering this improvement:

- The distance is calculated on the side of the vehicle, all the operations are performed in a special secure computing module;
- The distance is calculated on the side of the base station, while the vehicle actions are limited to sending requests and receiving answers.

### 4.1. Vehicle-Centered Approach

**Figure 7:**
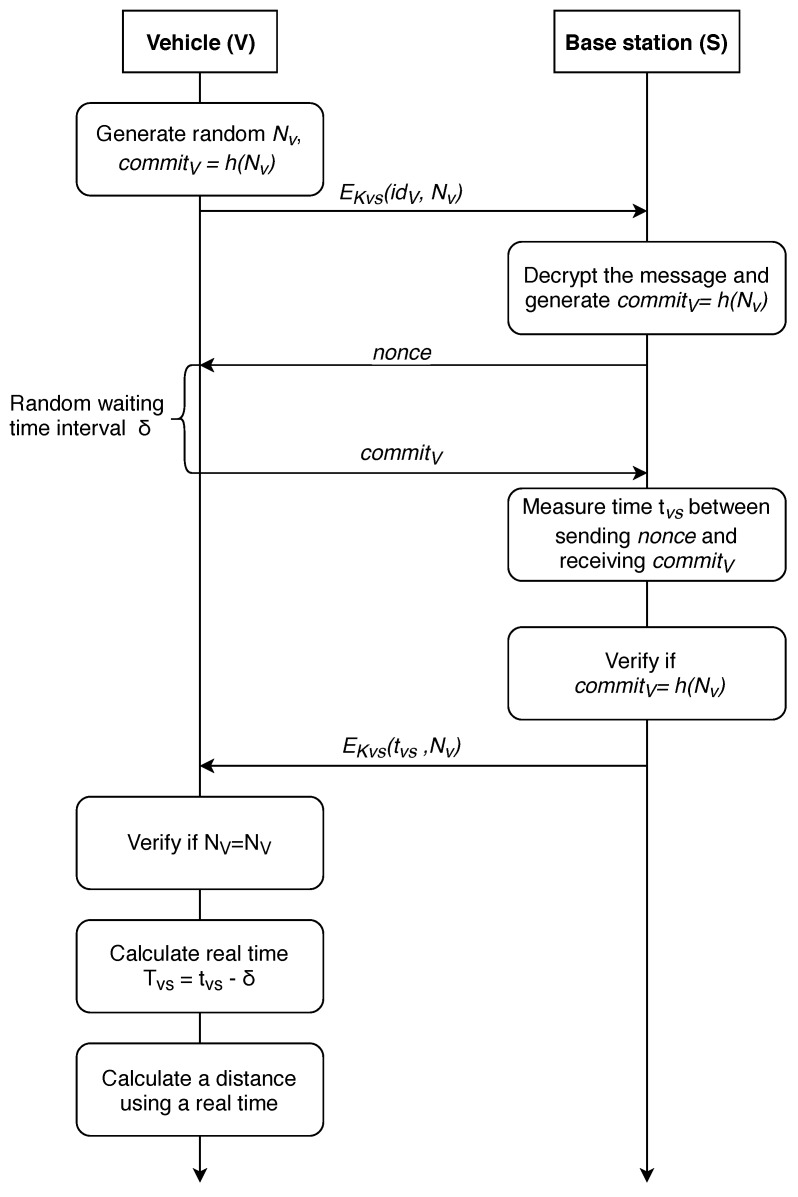
A distance-bounding P-AF-V protocol with the property of anonymity (calculations are made on the vehicle side).

First, we consider the case where the calculation is carried out on the vehicle side. In this case, the vehicle needs to contact at least three BSs, calculate the time of the signal traveling to the base station, and then, using verifiable multilateration method [63], calculate its coordinates. The P-AF-V protocol for exchanging information with the base station is shown in Figure 7.

After receiving the message nonce from the base station, the vehicle chooses a random number δ, which determines the delay in response to the base station, waits for this time interval and sends a commitment message to the base station afterwards. Thus, the base station does not know the random delay time chosen by the vehicle and, therefore, does not have an ability to determine the real time $t_{VS}$ of the signal passing to the vehicle and back.

Accordingly, the station transmits its miscalculated time of passing the signal $\bar{t_{VS}}=t_{VS}+\delta$ to the vehicle, which can, in turn, calculate the real time of the signal passing through it $t_{VS}=\bar{t_{VS}}-\delta$. It is worth noting that the vehicle must quickly obtain information from at least three different stations, calculate the distance to a particular station, and then calculate the coordinates.

### 4.2. Protocols of the Distance Determination Which Have the Property of Anonymity

**Figure 8:**
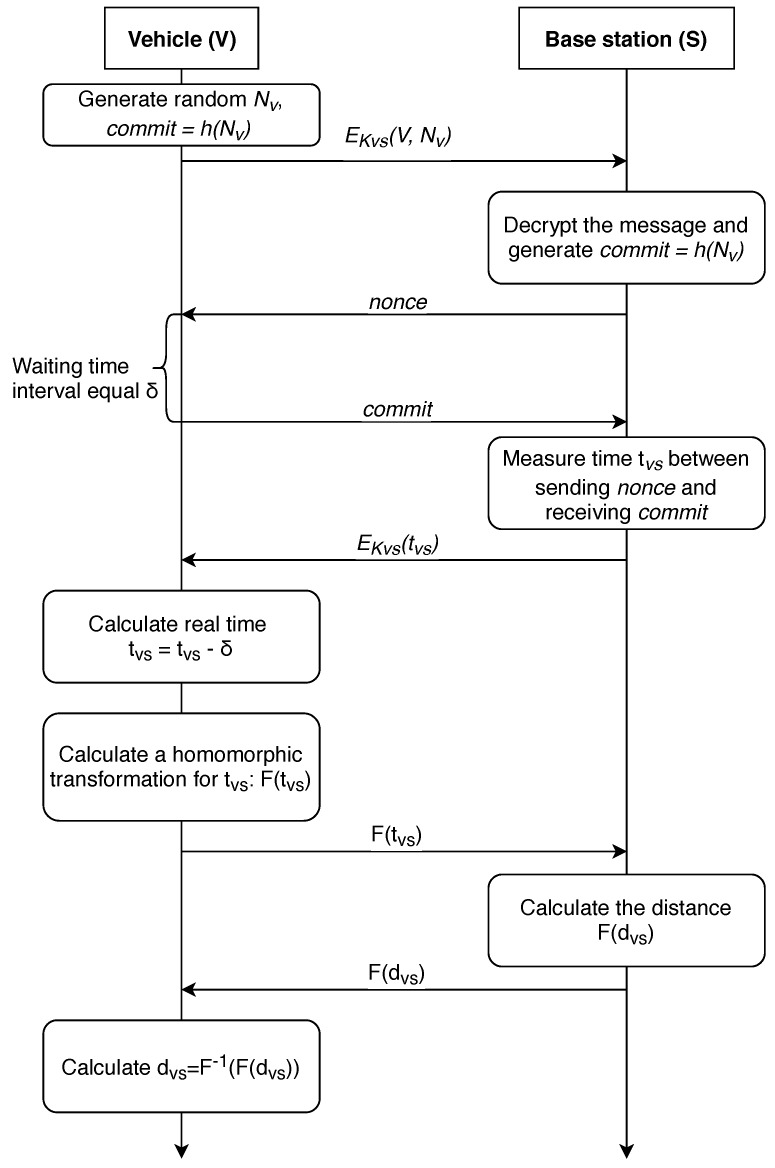
A distance-bounding P-AF-BS protocol with the property of anonymity (calculations are made on the base station side).

Now, we will consider the protocol where the calculation is carried out at the station side. The corresponding protocol is shown in Figure 8.

In this protocol, we use a fully homomorphic encryption (FHE) [88]. This technique is detailed in [89,90]. We repeat all the steps (excluding the last one) from the protocol of vehicle-side distance calculation, shifting this calculation to the side of the base station. Further, sending the result of the homomorphic transformation $F(t_{VS})$ to the base station, where the distance to the vehicle is calculated, or using the information received from other stations, its location is determined, the value $F(d_{VS})$ or the result of a homomorphic transformation of the coordinates of the car. After this, the received information is delivered to the vehicle. The use of a homomorphic transformation obfuscates the true values of $t_{VS}$ from the base station, and the vehicle determines the true value of the distance to a particular station or its location in this case. This allows for avoiding complex calculations at the vehicle side, shifting the calculation workload to the BS side.

Here, we compare the discussed protocols and also elaborate on the improved one. Further on, *n*—is a message length; λ—the system security parameter. Let’s consider our protocols and estimate three factors: number of operations, the storage space, and the complexity of the calculations as: Random number generation—O(1); Transmission—O(1); Hashing, Encryption (MD5)—O(n) [91]; Signature—O(n) [91]; Verification—O(n) [91]; Measurement—O(n) [92]; Calculation (subtraction)—O(log(n)) [92]; XOR—O(n) [93]; and Homomorphic encryption—$O(\lambda^{3.5})$ [94].

**Figure 9:**
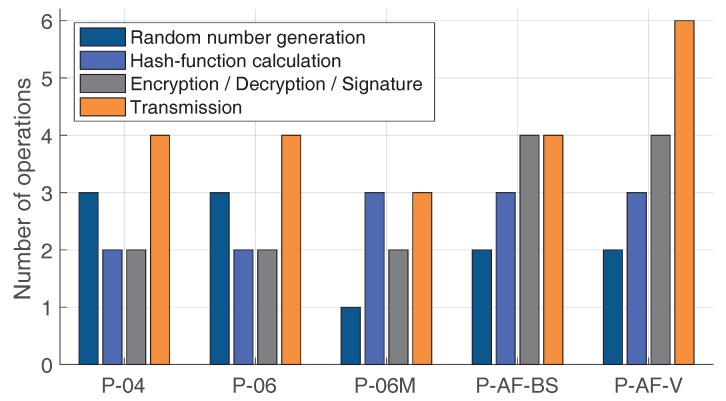
Number of different operations required per protocol.

From the Table 2 it can be seen that the less computationally expensive protocol is the improved distance-bounding one because there are less operations, as it is shown in Figure 9, and only one random nonce is stored. Of course, the last proposed protocol is the most difficult one which is mainly due to the introduced anonymity improvement.

## 5. Related Security and Privacy Threats

In this section, we list the attacks explicitly dangerous for location data exchange with another nodes. Note, we further elaborate not only on the attacks explicitly related to the exchange of the data between the vehicle and infrastructure node.

Attackers are mainly focusing on two main sides while penetrating ITS systems. First, to get unauthorized access based on weaknesses in social engineering and/or physical protection of infrastructure elements and onboard controllers. Second, to exploit the design of the security protocols and communications mainly in terms of message modification or replaying previously received messages [95]. Most of the attacks today are solved with Trusted Platform Module (TPM)-based protection, identity-based cryptography, and short-lived key certificates.

### 5.1. Security Threats

**Conventional and Infrastructure-Related Attacks**

*Man-in-the-middle attack (MITM):* (today also referred to as person-in-the-middle attack) is an active attack where an eavesdropper can intercept and modify fully or partly the positioning signaling between two vehicles or between a vehicle and the terrestrial infrastructure used for positioning, such as cellular signaling [96]. Increasing the security and authentication of the data signaling protocols is one solution to mitigate this attack type.

*Replay attack:* This attack is similar to the previous one. Here, the attacker can replay the messages traveling between vehicles or vehicles and infrastructure thus affecting the correctness of the positioning itself instead [97].

*Sybil Node (Rogue AP):* This attack and hardware/software unauthorized modifications are another active attacks, where BS or AP would transmit intentionally erroneous positioning-related data [98]. To cope with this attack type, outlier detection mechanisms need to be used [99].

*Jamming attack:* The attacker can be either a stationary or a moving jammer. Given the nature of the mobility of the vehicular networks, it is rational to have a moving jammer that tracks the desired node and causes regular interference. Utilizing spread spectrum techniques could assist in addressing this attack. As one of the options, the Frequency Hopping Spread Spectrum (FHSS) makes the incoherent signal period impulse-noise to the eavesdroppers. On the other hand, Direct Sequence Spread Spectrum (DSSS) symbolizes each data bit in the original signal by multiple bits in the transmitted signal [100] which is also a recommended technique to resist jamming.

*Passive Eavesdropping attack:* In this type of passive attack, an attacker can listen to the positioning signaling between two vehicles or between a vehicle and the infrastructure [101]. If the positioning signaling channel is strongly encrypted – this type of attack may be mitigated.

*Message modification attack:* This attack is targeted at altering the message during or after transmission (active attack) [102]. The adversary may wish to change the source or content of the message in terms of the position or time information that had been sent and saved in its device, in order to escape the consequences of a criminal/car accident event.

*Key and/or Certificate Replication:* This attack involves the system endorsement with a node of similar identity. The execution of the attack relies on the key management or certificates replacement, in order to forbid the identification and abuse the authorities [103].

While most of the security threats could be mitigated by employing the integration of stronger information security systems, privacy-related attacks could not be overcome by mere modifying the hardware/software side of the system.

**Attacks Related to Distributed Operation**

*Congestion attacks:* Generally, urban traffic congestion is considered as one of the major problems from the vehicular systems’ operation perspective. Modern cars are already available to report the congestion-related data either with integrated wireless modules or via hand-held user devices. Focusing on the first case, Vehicular Ad-Hoc Networks (VANETs) can provide timely information to the surrounding nodes which result in more effective route selection (around a sudden traffic jam) using the information obtained from other cars. From the information security perspective, the V2V radio connectivity of autonomous vehicles will offer attackers opportunities to combine multiple compromised vehicles into botnets of cars, which will lead to other serious security consequences [104].

From the infrastructure perspective, such attacks may potentially lead to incorrect traffic light signaling. As a result, the optimization algorithm running in the ITS cloud may operate based on the incorrect data, leading to congestion [95].

*Repudiation attacks:* This attack is an example of adequately tracking and recording the user actions, which can maliciously manipulate or fake the identification of new nodes [105]. This attack is used to modify the user’s information about the actions performed by the malicious and to register false data in log files. It can also be used to manipulate the master data in a similar pattern involving the messages. If this attack takes place, the data stored in the log files may be considered invalid or misleading.

*Routing Loop Attack:* In this type of attack, an internal attacker receives a message, updates it and sends it back to one of the previous forwarders (or the source) even if there is a better node in its routing table that is available to be the next forwarder according to the routing strategy. This attack aims to delay or prevent the delivery of a message [106]. The primary countermeasure against routing-related attacks is by utilizing Intruder Detection techniques and excluding the node from the network [107].

*Sinkhole Attack:* In this type of routing attack, the attacker (already being in the system, i.e., an internal one) attempts to announce the nodes in the network with a different location in its beacon messages followed by drops of any other packets [108]. It results in the poisoning of the routing tables and packet loss. The attacker may also be intellectual and analyze the captured packets while conventional one just affects the overall system operation in a harmful way.

*Wormhole Attack:* This type of routing attack corresponds to cases of two terminals having a link in between and they resend messages received by one of them to another [109]. Therefore, they can dominate on the routing path by replaying the valid beacons from other nodes. Attackers tend to dominate the connection so they can eavesdrop or share the network.

### 5.2. Privacy Threats

*Unauthorized use of location data and location-based services:* This threat could be considered as unauthorized use of tolled highways pretending to have a position of a neighbor vehicle which is not situated on a highway or unauthorized access in a car park [110].

*Disclosure of unwanted information:* As an example, consider a case such as if a person leaving the house empty for a long trip by car or by boat (which may enable house burglaries); how often an employee, supposed to be at the office, is visiting places by car (which may lead to loss of social reputation); how often a spouse is visiting places that he/she has never mentioned to his/her relatives (which may cause family crises), etc.

*Tracking malware applications:* A vehicle-installed software could contain malware to “steal” the tracking patterns of the users inside the vehicle, i.e., the user positions and speeds in time; such information, if sold to car dealers, could adversely affect the price of a vehicle, e.g., in function of the types of roads where vehicle and how often it was driven, etc.

*Scrambler attack:* An additional measure to achieve a higher level of anonymity in vehicular systems is the utilization of dynamically changing identifiers (from MAC to application layer), so-called pseudonyms [111]. The effectiveness of this approach, however, is clearly reduced if specific characteristics of the physical layer (e.g., in the transmitted signal) reveal the link between two messages with different pseudonyms. In contrast to other physical layer fingerprinting methods, it does not rely on potentially fragile features of the channel or the hardware but exploits the transmitted scrambler state that each receiver has to derive in order to decode a packet, making this attack extremely robust. The most straightforward solution is to employ a cryptographic pseudo-random number generator, possibly seeded by a large number of entropy sources in a vehicle (e.g., engine start time, sensors data, SNR, vehicles nearby, etc.). Another solution is the deployment of constant network-wide scrambler values.

As compared to security threats, privacy issues always had more involvement by the actual humans. One option to improve privacy in ITS systems is to utilize the solutions with strong anonymity properties [112,113] or frequently changing pseudonyms [114]. Some researchers foresee that Software-defined networking (SDN) will become an ultimate enabler for overcoming previously listed security and privacy problems of ITS [115,116] supporting both V2V and V2I scenarios.

## 6. Discussion and Future Perspectives

This section provides an overview of the future development of privacy strategies in positioning from EU regulations perspective in addition to conclusions of this work.

Recently introduced General Data Protection Regulation (GDPR) policy broadly states that *profiling* and processing the location information of an individual could be done concerning personal aspects which are naturally related to person’s movement and location data. According to [117], it could be done only when EU law regulations or the Member States demands or allows it, or “with the explicit consent of the data subject.” Thus, tracking and monitoring the data of the user’s vehicle also falls within this regulation. However, the definition of *personal location* data should be treated more carefully especially for cases when the *identifiability* of the said individual could be established. This way, the GDPR aspects related to the protection of personal data also should be taken into consideration while operating with personal location data.

In the ITS case and focusing on conventional GNSS systems, all the supported systems are controlled by the authorities, namely military for GPS and GLONASS and, in contrast, civilian Galileo in EU, which makes the implementation of GDPR more complicated. In order to overcome this issue, European Telecommunications Institute (ETSI) is actively developing a standard EN 303 413 [118] aiming to overcome the interoperability of those systems. However, the corresponding questions remain unclear from the telecommunications side especially while speaking about 5G [119]. Moreover, location-related data may be stored in the environments potentially not controlled by the operators themselves [120].

Industrial giants, such as Huawei, already rise the question on how to address the challenges of private positioning in future networks [121]. One of the fundamental principles to be followed is by following the GDPR Article 25, i.e., to implement a privacy-by-design approach to achieve privacy from the commencement of the system perspective.

Aiming to achieve the above-mentioned, we have shown different location estimation techniques for ITS scenario in this paper. We analyzed the existing protocols for the information exchange between the vehicle and the base station during the localization process. Subsequently, we presented an improved protocol for the data exchange, where much attention was paid to the security of signaling information transmission. Using the modified protocol, the vehicle can also rely on the location data without disclosing the identity, and thus the privacy could be generally improved.

In real life, the selection of the protocol highly depends on the application needs. If the system architect wants to have maximum anonymity, it is recommended to utilize the modified protocol, but it is the most difficult one for implementation. The optimal option is to use a protocol with a property of anonymity but BS-based. The complexity is not much higher compared to others, and there are no complex operations. 

## Figures and Tables

**Table 1:** Key constructs utilized in this work.

Construct	Description
*V*	Target vehicle
*S*	Target base station
$N_{V},N_{V}^{\prime },N_{S}$	Random nonces
h(x)	Hashing function
$id_{x}$	Unique identification number
$K_{VS}$	Pairwise symmetric key
$sig_{K_{VS}}\left(x\right)$	Signature by $K_{VS}$
$d_{VS}$	Distance between nodes
$t_{VS}$	Time between message exchange
σ	Delay in response to the base station
$\bar{t_{VS}}$	Miscalculated time of passing the signal
F(x)	Homomorphic transformation

**Table 2:** The complexity evaluation.

Protocol	Storage Space	Complexity
Protocol-2004 (P-04)	2 random nonces; Hash value	5O(n)+8O(1)
Protocol-2006 (P-06)	2 random nonces; Hash value; Symmetric keys	5O(n)+8O(1)
Modified Protocol-2006 (P-06-M)	Random nonce; Hash value; Symmetric keys	5O(n)+5O(1)
BS-based anonymity-focused protocol (P-AF-BS)	Random nonce; Hash value; Symmetric keys; Interval sigma	9O(n)+7O(1)
Vehicle-based anonymity-focused protocol (P-AF-V)	Random nonce; Hash value; Symmetric keys; Interval sigma	$2O\left(\lambda^{3.5}\right)+10O\left(n\right)+9O\left(1\right)$

## Abbreviations

The following abbreviations are used in this manuscript:

5G	5th generation cellular networks
AOA	Angle of Arrival
AP	Access Point
BDS	BeiDou Navigation Satellite System
BLE	Bluetooth Low Energy
BSID	Base station identificator
CP	Cooperative positioning
DSRC	Dedicated Short Range Communications
DOS	Denial of Service Attack
D2D	Device-to-Device communications
EN	European Union
ETSI	European Telecommunications Institute
FHE	Fully homomorphic encryption
GNSS	Global Navigation Satellite System
GDPR	General Data Protection Regulation
GPS	Global Positioning System
ITS	Intelligent Transportation Systems
ITS-G5	Intelligent Transport Systems operating in the 5 GHz frequency band
LoS	Lines of sight
LPWA	Low-Power Wide-Area Wireless Technology
LSP	Location Solution Provider
LIDAR	Light Detection and Ranging
MANET	Mobile ad hoc network
MEO	Medium Earth orbit
MITM	Man-in-the-middle attack
MIMO	Multiple Input Multiple Output
NR	New radio
RSS	Received signal strength
RSSI	Received signal strength indicator
TDOA	Time difference of arrival
TPM	Trusted Platform Module
TOA	Time of arrival
VANET	Vehicular Ad-Hoc Network
V2I	Vehicle-to-infrastructure paradigm
V2V	Vehicle-to-vehicle paradigm
V2X	Vehicle-to-everything paradigm
XOR	Exclusive OR operation
